# FAF1 mediates necrosis through JNK1-mediated mitochondrial dysfunction leading to retinal degeneration in the ganglion cell layer upon ischemic insult

**DOI:** 10.1186/s12964-018-0265-7

**Published:** 2018-09-10

**Authors:** Changsun Yu, Bok-seok Kim, Minyoung Park, Yun-Ju Do, Young-Yun Kong, Eunhee Kim

**Affiliations:** 1 Department of Biological Sciences, Chungnam National University, 99 Daehak-ro, Yuseong-gu, Daejeon, 34134 South Korea; 2 School of Biological Science, Seoul National University, 1 Gwanak-ro, Gwanak-gu, Seoul, 08826 South Korea; 3 MOGAM Institute for Biomedical Research, 93, 30beon-gil, Ihyeon-ro, Gilheung-gu, Yongin-si, Gyeonggi-do 16924 South Korea; 4 BeyondBio Inc., Daejeon BioVenture Town, 1662, Yuseong-daero, Yuseong-gu, Daejeon, 34134 South Korea

**Keywords:** FAF1, JNK1, Ischemic death, Retina

## Abstract

**Background:**

Aberrant cell death induced by ischemic stress is implicated in the pathogenesis of ischemic diseases. Fas-associated factor 1 (FAF1) has been identified as a death-promoting protein. This study demonstrates that FAF1 functions in death signaling triggered by ischemic insult.

**Methods:**

The expression changes of FAF1 and phophorylated JNK1 were detected by Western blotting. Immunoprecipitation was employed to investigate protein-protein interaction. We determined the cell death using flow cytometry and lactate dehydrogenase release measurement. To validate the death-promoting role of FAF1 in the retina, we generated conditional retinal FAF1 knockout mice. We used hematoxylin and eosin staining to detect retinal cell death in retinal ganglion cell layer.

**Results:**

FAF1 was found to function upstream of c-Jun N-terminal kinase 1 (JNK1), followed by mitochondrial dysregulation and necrotic cell death processes upon ischemic insult. We investigated whether FAF1 is involved in the pathogenesis of ischemic diseases using a retinal ischemia model. Indeed, FAF1 potentiated necrosis through JNK1 activation upon ischemic stress in retinal cells demonstrating retinal ganglion–like character. Conditional FAF1 depletion attenuated JNK1 activation in the retinas of Dkk3-Cre;*Faf1*^flox/flox^ mice and ameliorated death of retinal cells due to elevated intraocular pressure (IOP).

**Conclusions:**

Our results show that FAF1 plays a key role in ischemic retinal damage and may be implicated in the pathogenesis of retinal ischemic disease.

**Electronic supplementary material:**

The online version of this article (10.1186/s12964-018-0265-7) contains supplementary material, which is available to authorized users.

## Background

Ischemic stress results from sudden blood vessel occlusion by thrombus formation or embolism and consequent, nearly immediate loss of oxygen and glucose in the affected tissue [1]. Ischemic stress-induced cell death is implicated in the pathogenesis of diverse diseases. Ischemic stress produces either an apoptotic or a necrotic phenotype depending on the intracellular ATP level [1–4]. However, recent studies indicate that necrosis predominates over apoptosis upon ischemic insult because intracellular ATP is rapidly exhausted due to the limited oxygen and glucose [1, 2, 4–7].

Retinal ischemia model is a useful system to investigate underlying molecular mechanisms because of easier accessibility of the retina without losing the features of neuronal degeneration [8]. Moreover, retinal ischemia is implicated in diverse ocular diseases such as age-related macular degeneration, diabetic retinopathy and glaucoma [9]. Retinal ganglion cells (RGCs) have high metabolic demand to maintain membrane potential and to execute neurotransmission, demonstrating particular vulnerability to ischemic insult [10, 11]. c-Jun N-terminal kinase (JNK) activation was observed in RGCs of retinal ischemia animal models and glaucoma patients [12, 13]. Moreover, inhibition of JNK activation protected RGCs against retinal ischemic stress. Collectively, these data emphasize that JNK activation is a key process in the ischemic death of RGCs [14].

JNK activation often proceeds to mitochondrial dysfunction through JNK translocation to the mitochondria [15, 16]. Translocated JNK weakens mitochondrial bioenergetics and increases mitochondrial membrane permeability, resulting in cell death [15]. Additionally, blockade of JNK translocation to mitochondria protects cells against 6-hydroxydopamine-induced cytotoxicity [17]. Moreover, recent studies have revealed that mitochondrial resident proteins, such as Bcl-X_L_, myeloid cell leukemia sequence 1 (Mcl-1), and SH3BP5 (Sab), interact with JNK and regulate mitochondrial functions [18–20]. Taken together, the interaction between JNK and mitochondria seems to be crucial for regulating cell death.

The retinal ganglion cell line (RGC5) has been widely used as a cellular model to investigate the molecular mechanism of pathogenesis of retinal ischemia [21–23]. However, RGC5 cells are controversial regarding their origin and identity as ganglion cells because of their expression of markers characteristic to photoreceptor cells [24, 25]. In contrast, recent studies report that RGC5 cells retain characteristics of retinal ganglion cells. RGC5 cells express diverse RGC-specific markers such as Brn3b, Brn3c, Thy-1, γ-synuclein, NeuN and Opn1mw [26]. Moreover, overexpression of OPTN mutant found in the glaucoma patients selectively induced cell death of RGC5 cells but not other cells including HeLa, COS-7, Neuro2a and SH-SY5Y cells, supporting for the idea that RGC5 cells retain RGC-like properties [26]. Therefore, RGC5 seems to be a useful measure for elucidating the molecular mechanism underlying retinal ischemia to a certain extent considering the absence of authentic retinal ganglion cell line.

Certain death proteins, such as p53, B-cell lymphoma 2 (Bcl-2), and Bcl-X_L_, operate under distinctive mechanisms depending on the cellular oxygen level. p53 participates in the cell death process in response to both ischemic and oxidative stresses. However, the mechanisms through which p53 acts are different: p53 accumulates and displays quantitative regulation under ischemia, whereas it is phosphorylated and demonstrates qualitative regulation under oxidative stress [27]. Similarly, Bcl-2 and Bcl-X_L_ expression levels decrease during ischemia but increase in response to oxidative stress [28].

Fas-associated factor 1 (FAF1) has been identified as a death-promoting protein [29]. FAF1 participates in Fas-induced apoptosis as a member of the death-inducing signaling complex and regulated necrosis through activation of poly(ADP-ribose)polymerase 1 (PARP1) [30, 31]. In addition, FAF1 suppresses the NF-κB pathway [32, 33] and arrests the cell cycle, indicating it is a tumor suppressor [34]. FAF1 also regulates the chaperone activity of Hsp70, degradation of endoplasmic reticulum-associated substrates, and the innate immunity signaling pathway [35–38]. Our previous study showed that FAF1 contributes to cell death through PARP1 activation upon oxidative stress in dopaminergic neurons [30, 31]. This study investigated how FAF1 functions under ischemic condition.

In the current study, we report that FAF1 plays an essential role in ischemic stress-induced cell death by contributing to JNK1 activation. FAF1 depletion suppressed ischemic stress-induced JNK1 activation and robustly protected cells from ischemic cell death. Collectively, our results demonstrate an important contribution of FAF1 to the progression of retinal ischemic diseases.

## Methods

### Reagents and antibodies

The following reagents were obtained commercially. Rabbit anti-caspase-3, rabbit anti-P-JNK, and rabbit anti-P-c-Jun antibodies were from Cell Signaling Technology (Danvers, MA, USA). Mouse anti-Flag, mouse anti-HA, mouse anti-β-actin antibodies, Nec-1, DPQ, PI, and 50% glutaraldehyde were from Sigma-Aldrich (Saint Louis, MO, USA). The mouse anti-FAF1 antibody has been described previously [31]. Mouse anti-COX IV, horseradish peroxidase (HRP)-conjugated anti-mouse, HRP-conjugated anti-rabbit, and fluorescein (FITC)-conjugated anti-mouse antibodies and 4′,6-diamidino-2-phenylindole (DAPI) were from Thermo Fisher Scientific Inc. (Rockford, IL, USA). Mouse anti-JNK1 antibody and annexin V were from BD Biosciences (San Jose, CA, USA). Rabbit anti-GFP antibody was from Santa Cruz Biotechnology (Dallas, TX, USA). z-VAD-fmk was from Millipore (Darmstadt, Germany). SP600125 was from Tocris Bioscience (Minneapolis, MN, USA).

### Cell culture and transfection

MEFs were cultured in a humidified atmosphere (37 °C, 5% CO_2_) in Dulbecco’s modified Eagle’s medium (DMEM, WelGENE, Daegu, Korea) supplemented with 10% fetal bovine serum (Atlas Biologicals, Fort Collins, CO, USA) and 1% penicillin/streptomycin (Thermo Fisher Scientific, Inc.). RGC5 cells were cultured in DMEM supplemented with 10% fetal bovine serum under the same condition as MEFs. MEF and RGC5 cells were transfected with the indicated plasmids using BioT reagent (Morganville Scientific, Morganville, NJ, USA).

### Gene silencing with siRNA

Small interfering RNA (siRNA) oligonucleotides were purchased from Bioneer (Daejeon, Korea). The target sequences for the siRNA against FAF1 and JNK1 were siFAF1 (5′- CUGACAAAGGACGCCAACA -3′) and siJNK1 (5′- CUGGAUAUAGCUUUGAGAA -3′).

*Faf1*^*gt/gt*^ MEFs and RGC5 cells were transfected with 200 nM of siRNA or scRNA using Lipofectamine RNAi MAX reagent, respectively, (Thermo Fisher Scientific Inc.) according to the manufacturer’s instructions.

### Oxygen glucose deprivation

The MEF and RGC5 cell media were replaced with glucose-free deoxygenated medium containing HEPES (10 mM), NaCl (116 mM), KCl (5.4 mM), NaH_2_PO_4_ (0.8 mM), sodium bicarbonate (25 mM), sucrose (25 mM), CaCl_2_ (1.8 mM), and phenol red (0.04%; pH 7.3) and incubated in an anaerobic chamber (Thermo Fisher Scientific Inc.) with a CO_2_ (5%), H_2_ (10%) and N_2_ balance at 37 °C for the indicated times.

### Co-immunoprecipitation and immunoblot analysis

MEF and RGC5 cells were lysed in mammalian cell lysis buffer (50 mM Tris-HCl; pH 8.0, 150 mM NaCl, 1 mM EDTA, 1% Nonidet P-40, 0.4 mM phenylmethylsulfonyl fluoride). The protein levels were quantified using a Bio-Rad Protein Assay Kit (Bio-Rad, Hercules, CA, USA). Co-immunoprecipitation was performed with the indicated antibodies and protein A/G Sepharose (Santa Cruz Biotechnology). Samples were separated by SDS-PAGE and transferred to nitrocellulose membranes. The membranes were blocked with 5% skim milk and incubated with suitable primary antibodies. After incubation, the membranes were incubated with HRP-conjugated secondary antibodies. The protein bands were detected using a Chemiluminescence Detection Kit (AbFrontier, Seoul, Korea).

### Lactate dehydrogenase (LDH) release assay

The cells were seeded into 96-well plates (MEFs, 10,000 cells per well) and incubated for 12 h. The MEFs were exposed to oxygen glucose deprivation for the indicated times. Cell death was assessed by the release of LDH into the extracellular medium, which was measured with a Cytotoxicity Detection Kit (Roche, Basel, Switzerland).

### Caspase-3 activity assay

MEFs were exposed to oxygen glucose deprivation or STS for the indicated times. Next, caspase-3 activity was measured using a Caspase-3 Colorimetric Assay Kit (Biovision, Milpitas, CA, USA) according to the manufacturer’s protocol. The absorbance at 450 nm was measured using a VICTOR microplate reader (PerkinElmer, Norwalk, CT, USA).

### Measurement of mitochondrial potential

MEFs were treated with oxygen glucose deprivation for 5 h and then harvested. Mitochondrial membrane depolarization was measured using a Muse MitoPotential Kit (Millipore). Briefly, cells were incubated with Muse MitoPotential dye for 20 min in a 37 °C CO_2_ incubator. Then, mitochondrial membrane potential changes were determined with a Muse analyzer (Millipore).

### Measurement of mitochondrial ROS production

MEFs were treated with oxygen glucose deprivation for 5 h and then harvested. Mitochondrial ROS production was measured using a Guava easyCyte flow cytometer (Millipore). Briefly, cells were incubated with MitoSOX Red mitochondrial superoxide indicator (Thermo Fisher Scientific Inc.) for 10 min in a 37 °C CO_2_ incubator. Then, mitochondrial ROS production was determined with the Guava easyCyte flow cytometer and quantified using InCyte software (Millipore).

### Subcellular fractionation

Subcellular fractionation was performed using a Mitochondria Isolation Kit with some modifications (Thermo Fisher Scientific Inc.). In brief, MEFs were suspended in commercially supplied mitochondria Isolation Reagent A (Thermo Fisher Scientific Inc.) and homogenized by passaging through a 26-gauge syringe needle 150 times. The lysates were centrifuged at 720×g for 10 min. After the supernatant was transferred to a new tube, it was centrifuged at 12,000×g for 10 min. The supernatant was used as the cytoplasmic fraction, and the pellet was washed twice with the same buffer and used as the mitochondrial fraction.

### Flow cytometry

MEFs and RGC5 cells were treated with oxygen glucose deprivation for the indicated times. Cells were harvested and stained with PI at a final concentration of 5 μg/ml. Cell death was measured using a Guava easyCyte flow cytometer (Millipore). In another set of experiments, oxygen glucose deprivation- or STS-treated MEFs were harvested and washed using annexin V buffer provided by the supplier (BD Biosciences) and then stained with annexin V. Next, PI was added at a final concentration of 5 μg/ml. The cells were then evaluated using a Guava easyCyte flow cytometer and quantified using InCyte software (Millipore).

### Mice

Twelve-week-old male C57BL/6 J (Central Lab Animal Inc., Seoul, Korea), Dkk3*-*Cre;*Faf1*^*+/+*^ and Dkk3*-*Cre;*Faf1*^*flox/flox*^ mice were used for the in vivo experiments. All mice were maintained in the animal facility of Chungnam National University (Daejeon, Korea) and acclimatized to a light schedule of alternating 12 h periods of light and dark with free access to food and water. All animal studies were conducted in accordance with the institutional guidelines for the care and use of laboratory animals.

### FAF1 conditional knockout mouse generation and breeding

To create a FAF1 conditional knockout mouse, we used the Cre-loxP system [39]. The targeting vector contained intron 3, exon 4 and intron 4 of the *Faf1* gene, in which two loxP sites were inserted into introns 3 and 4. The targeting vector introduced the loxP sites into the *Faf1* locus through recombination (Fig. 7a)*.* Retina-specific DNA sequence deletion of the *Faf1* gene between the two loxP sites was achieved by breeding Dkk3-Cre mice, based on the predominant expression of Dkk3 in retinal progenitor cells [40], for at least 3 generations prior to the study. Dkk3-Cre mice were a gift from Dr. Jin Woo Kim (Korea Advanced Institute of Science and Technology, Daejeon, Korea). Genotyping was performed via PCR using DNA extracted from tail clippings with a Direct PCR (Tail) Kit (Viagen Biotech Inc., Los Angeles, CA, USA), and the relevant primer sequences are shown in Additional file 1: Table S1.

### Establishment of the mouse model of retinal ischemia

The experimental protocol was approved by the experimental animal ethics committee of Chungnam National University. The mice were anesthetized by intraperitoneal injection of a mixture of 10 mg/kg tiletamine-zolazepam (Zoletil 50, Virbac, Milan, Italy) with 1 mg/kg xylazine (Rompun, Bayer, Leverkusen, Germany). The middle of the anterior chamber of the experimental eye was cannulated with a 30-gauge needle connected to a pressure device [41]. The IOP was maintained at 140 mmHg for 60 min. In the contralateral eye, the anterior chamber was cannulated with a 30-gauge needle without elevation of the IOP. During the procedure, the temperature of the animal was maintained at 37 °C with a heating pad.

### Protein extraction from the retina

Retinas from enucleated eyes of mouse were homogenized in lysis buffer as stated above. Homogenates were centrifuged at 12,000×g for 10 min at 4 °C. Total protein levels were quantified using a Bio-Rad Protein Assay Kit (Bio-Rad). Samples were subjected to immunoblot analysis.

### Hematoxylin and eosin staining

Enucleated eyes of mouse were prefixed in 4% glutaraldehyde for 20 min and then lens were removed. Next, the samples were fixed in 4% glutaraldehyde for 12 h and embedded in paraffin wax. After embedding, retinal cross sections were performed with a thickness of 5 μm. The slides were deparaffinized, rehydrated and then stained with hematoxylin and eosin (HE) using a standard protocol [42]. After HE staining, the slides were dehydrated and mounted on coverslips.

### Statistical analysis

At least 3 independent experiments were carried out in vitro and in vivo. All data are expressed as the mean ± S.E.M. Statistical comparisons were performed using Student’s t-test or one-way analysis of variance (ANOVA) followed Tukey’s post hoc analysis using SPSS software (Statistic version 22; IBM Inc., Chicago, IL, USA). A *p*-value less than 0.05 was considered to indicate a significant difference among groups.

## Results

### JNK1 contributes to necrosis upon ischemic stress induced by oxygen-glucose deprivation in MEFs

**Fig. 1:**
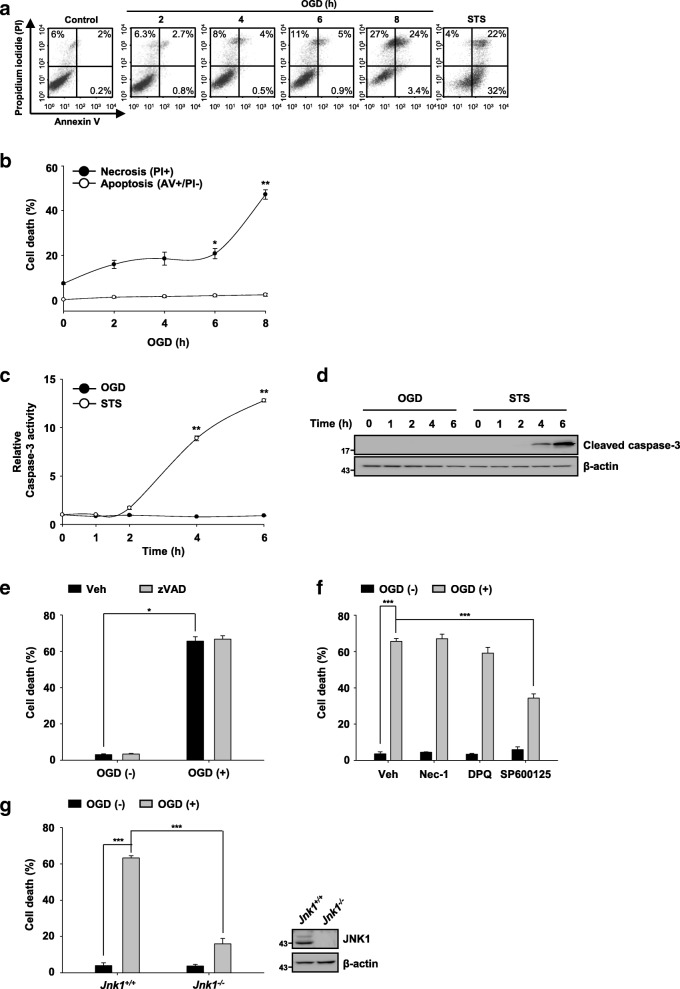
JNK1 contributes to necrosis upon ischemic stress induced by oxygen-glucose deprivation in MEFs. **a** MEFs were treated with oxygen glucose deprivation for the indicated times and/or 1 μM staurosporine (STS) for 12 h. The type of cell death was determined by flow cytometry using double staining with annexin V and PI. **b** The graph shows the quantitative results of the flow cytometry analysis. **c** and **d** MEFs were treated with oxygen glucose deprivation or 1 μM STS for the indicated times (*n* = 3). Caspase-3 activity was measured using fluorometric assay (*n* = 3) (**c**) and western blot analysis (**d**). **e** MEFs were untreated or treated with oxygen glucose deprivation for 8 h in the presence or absence of zVAD-fmk (50 μM), and cell death was determined by measuring PI uptake using a flow cytometer (*n* = 3). **f** MEFs were pretreated with Nec-1 (50 μM), DPQ (30 μM), or SP600125 (20 μM) for 30 min and then with oxygen glucose deprivation for 8 h in the presence of the individual compounds. Cell death was detected by flow cytometry (*n* = 3). **g** Left panel: Jnk1+/+ and Jnk1−/− MEFs were untreated or treated with oxygen glucose deprivation for 8 h. Cell death was detected using flow cytometry (*n* = 3). Right panel: representative immunoblots show the JNK1, FAF1 and β-actin expression levels. The data (**b**, **c**, **e**-**g**) are expressed as the mean ± S.E.M. of three independent experiments. Statistical comparisons were evaluated using ANOVA followed by Dunnett’s T3 (**b, c,** and **e**) and Tukey’s HSD (**f** and **g**) post hoc analysis. ***p < 0.001, **P < 0.01, and *P < 0.05

Oxygen glucose deprivation is commonly used to study ischemic cell death in vitro [43]. To investigate the type(s) of cell death upon ischemic stress elicited by oxygen glucose deprivation, we first performed flow cytometric analysis using propidium iodide (PI) and annexin V staining. As shown in Fig. 1a and b, exposure of mouse embryonic fibroblasts (MEFs) to oxygen glucose deprivation increased the PI single-positive and PI/annexin V double-positive cell populations in a time-dependent manner. Moreover, caspase-3 activation was not observed upon ischemic stress induced by oxygen glucose deprivation in MEFs (Fig. 1c and d). Furthermore, zVAD-fmk, a pan-caspase inhibitor, failed to protect MEFs against oxygen glucose deprivation (Fig. 1e). In contrast, treatment with the well-known apoptosis inducer staurosporine increased the annexin V single-positive and PI/annexin V double-positive cell population and caspase-3 activation in MEFs (Fig. 1a, c, and d). These results were consistent with previous studies in which the apoptotic machinery was intact in MEFs [44]. These data indicate that ischemic stress elicited by oxygen glucose deprivation induces non-apoptotic cell death in MEFs.

Diverse proteins, including receptor-interacting protein kinase 1 (RIPK1), PARP1, and JNK1, are implicated in ischemic stress-induced cell death [45–47]. Therefore, we examined whether these proteins were involved in oxygen glucose deprivation-induced necrosis in MEFs (Fig. 1f). The JNK1 inhibitor SP600125 significantly reduced oxygen glucose deprivation-induced necrosis in MEFs. However, a RIPK1 inhibitor (necrostatin-1, Nec-1) and PARP1 inhibitor DPQ did not affect oxygen glucose deprivation-induced MEF necrosis. Moreover, JNK1-knockout (*Jnk1*^*−/−*^) MEFs showed robust resistance against oxygen glucose deprivation effects (Fig. 1g). Therefore, we wondered whether the apparent major role of JNK1 in necrosis was limited to oxygen glucose deprivation or applied to other ischemic insults. To address this question, we investigated whether exposure to chemical hypoxia (CH) through metabolic inhibition and TNFα-cycloheximide-z-VAD (TCZ), other types of ischemic stresses, could induce JNK1-dependent necrotic cell death in MEFs. We observed that exposure of MEFs to CH- or TCZ-induced necrotic cell death (Additional file 2: Figure S1a-c) and JNK1 depletion protected MEFs from CH and TCZ, indicating that JNK1 also participates in the ischemic stresses including CH and TCZ (Additional file 2: Figure S1d and S1e). In contrast, RIPK1 inhibitor (Nec-1) demonstrated robust protection against TCZ, showing that RIPK1 activation is involved in the TCZ-induced death. However, Nec-1 did not protect MEFs against CH (Additional file 2: Figure S1f). Collectively, our data demonstrate that JNK1 predominantly contributes to necrosis in MEFs upon ischemic stress.

### FAF1 is required for JNK1-dependent necrosis upon ischemic stress

**Fig. 2:**
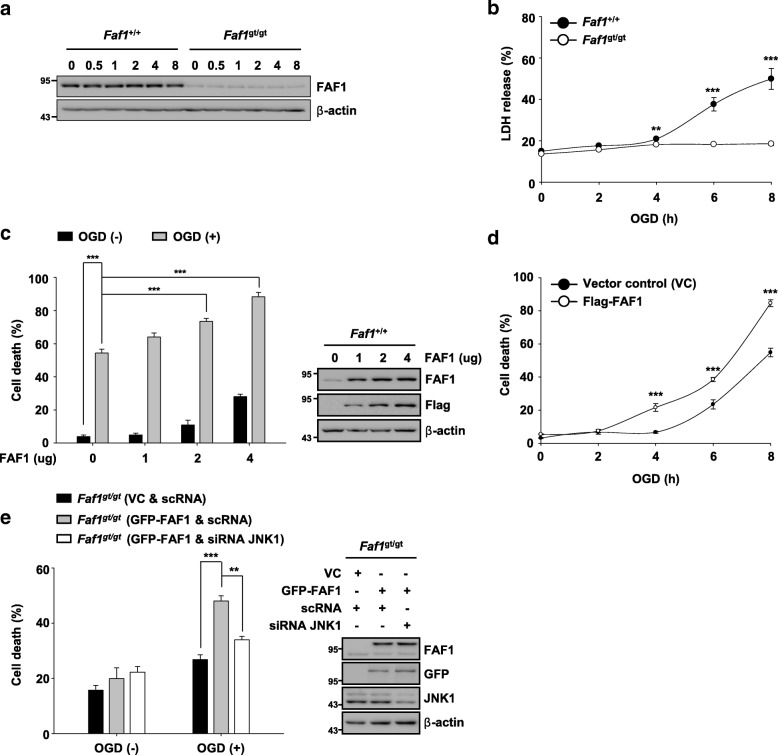
FAF1 is essential for JNK1-dependent necrosis upon ischemic stress. **a** *Faf1*^+/+^ and *Faf1*^gt/gt^ MEFs were treated with oxygen glucose deprivation for the indicated times, and then, cell lysates were immunoblotted using anti-FAF1 antibody. **b** *Faf1*^+/+^ and *Faf1*^gt/gt^ MEFs were treated with oxygen glucose deprivation for the indicated times. Cell death was determined by measuring LDH release (*n* = 3). **c** Left panel: *Faf1*^+/+^ MEFs were transfected with the indicated concentrations of Flag-FAF1 plasmid. At 36 h after transfection, the cells were untreated or treated with oxygen glucose deprivation for 8 h. Cell death was determined by measuring PI uptake using a flow cytometer (*n* = 3). Right panel: representative immunoblots showing the Flag, FAF1 and β-actin expression levels. **d** *Faf1*^+/+^ MEFs were transfected with vector control (VC) or Flag-FAF1 plasmids (4 μg). At 36 h after transfection, the cells were treated with oxygen glucose deprivation for the indicated times. Cell death was determined by flow cytometry (*n* = 3). **e** Left panel: *Faf1*^gt/gt^ MEFs were transfected with scrambled-siRNA (scRNA) or siRNA JNK1 (siJNK1) for 36 h, and subsequently transfected with VC or GFP-FAF1 plasmids (4 μg). At 24 h after transfection, the cells were untreated or treated with oxygen glucose deprivation for 6 h. Cell death was determined by flow cytometry (*n* = 3). Right panel: representative immunoblots showing the FAF1, GFP, JNK1 and β-actin expression levels. The data (**b** - **e**) are expressed as the mean ± S.E.M. of three independent experiments. Statistical comparisons were evaluated using ANOVA followed by Tukey’s HSD (**b** - **e**) post hoc analysis. ***P < 0.001, and **P < 0.01

FAF1 plays a key role in necrosis as well as in apoptosis [30, 31, 48]. Moreover, FAF1 activates JNK1 in response to certain stimuli, such as oxidative stress and heat shock [30, 35]. Therefore, we investigated whether FAF1 participates in JNK1-dependent necrosis upon ischemic stress. First, we examined the FAF1 expression levels in wild-type (*Faf1*^+/+^) and FAF1-depleted (*Faf1*^gt/gt^) MEFs upon ischemic stress induced by oxygen glucose deprivation. The FAF1 expression levels in *Faf1*^+/+^ and *Faf1*^gt/gt^ MEFs were not altered throughout the assessed time period under oxygen glucose deprivation conditions (Fig. 2a). However, depletion of FAF1 in MEFs conferred robust protection against oxygen glucose deprivation-induced cell death (Fig. 2b). FAF1 depletion in MEFs also rendered MEFs resistant to CH and TCZ (Additional file 3: Figure S2). In contrast, FAF1 overexpression potentiated oxygen glucose deprivation-induced cell death in a dose- and time-dependent manner (Fig. 2c and d). Furthermore, reconstitution of FAF1 in *Faf1*^gt/gt^ MEFs restored sensitivity to ischemic stress induced by oxygen glucose deprivation (Fig. 2e). Next, we examined whether knockdown of JNK1 by small interfering RNA (siRNA) in *Faf1*^gt/gt^ MEFs affected cellular sensitivity to oxygen glucose deprivation when FAF1 expression was restored. We found that siRNA-mediated JNK1 knockdown in *Faf1*^gt/gt^ MEFs attenuated cell death induced by oxygen glucose deprivation even under restored FAF1 expression, indicating that oxygen glucose deprivation-induced cell death via FAF1 depends on JNK1 (Fig. 2e). Taken together, our results indicate that FAF1 participates in the JNK1-dependent necrotic cell death upon ischemic stress.

### FAF1 interacts with JNK1 upon ischemic stress

**Fig. 3:**
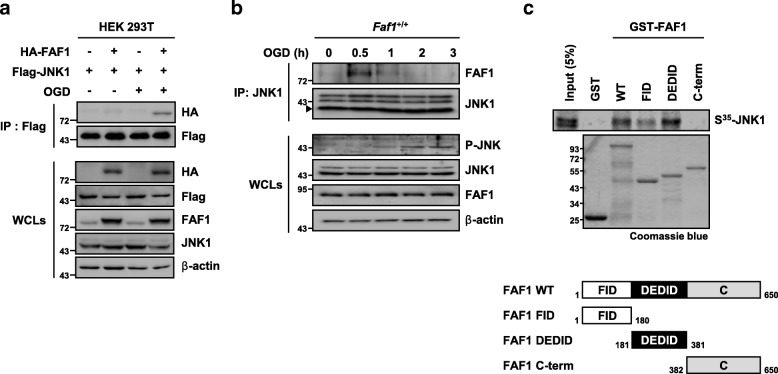
FAF1 interacts with JNK1 upon ischemic stress. **a** HEK 293 T cells were transfected with the indicated combinations of HA-FAF1 (1 μg) and Flag-JNK1 plasmids (1 μg). At 48 h after transfection, the cells were untreated or treated with oxygen glucose deprivation for 30 min. Whole cell lysates were immunoprecipitated with anti-Flag antibody, followed by immunoblotting with the indicated antibodies. **b** *Faf1*^+/+^ MEFs were treated with oxygen glucose deprivation for the indicated times. The cell lysates were then immunoprecipitated with anti-JNK1 antibody. The interaction was determined by immunoblotting with the indicated antibody. Filled triangle denotes a non-specific band (**c**) Upper panel: GST pull-down assay in which in vitro-translated ^35^S-labeled JNK1 was incubated with GST and GST-FAF1 fragments, and the in vitro-translated proteins present in the input (5%) or those associated with FAF1 were detected by autoradiography. Lower panel: Schematic diagram of full length and truncated FAF1 constructs. FID, Fas-interacting domain; DEDID, death effecter domain-interacting domain; C-term, c-terminal domain

Based on the finding that FAF1 participates in JNK1-dependent necrosis, we examined whether FAF1 interacts with JNK1 upon ischemic stress. In a co-immunoprecipitation assay, we found that the complex comprising HA-FAF1 and Flag-JNK1 was clearly detected in HEK 293 T cells in response to oxygen glucose deprivation (Fig. 3a, Additional file 4: Figure S3). We also confirmed that binding of endogenous FAF1 to JNK1 was only transiently observed and not apparent 2 h after oxygen glucose deprivation, indicating that FAF1 interacts with JNK1 in an ischemic stress-dependent manner (Fig. 3b). Given that FAF1 interacts with JNK1, we next examined which domain of FAF1 was responsible for binding to JNK1 using in vitro-translated ^35^S-labeled JNK1. A GST pull-down assay using truncated FAF1 mutants showed that the death effector domain-interacting domain (DEDID) of FAF1 strongly interacted with JNK1, and the Fas-interacting domain (FID) of FAF1 showed weak binding to JNK1 (Fig. 3c). This JNK1 binding pattern, encompassing two N-terminal domains of FAF1, has also been observed in IKKβ [49].

### FAF1 positively regulates JNK1 activity upon ischemic stress

**Fig. 4:**
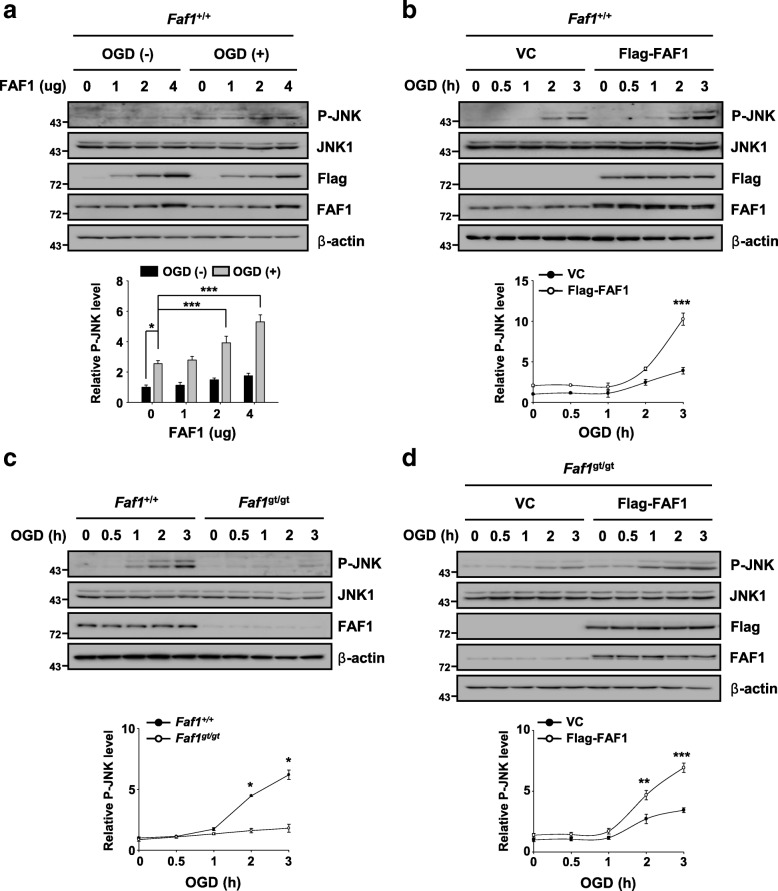
FAF1 positively regulates JNK1 activity upon ischemic stress. **a** Upper panel: *Faf1*^+/+^ MEFs were transfected with the indicated concentrations of Flag-FAF1 plasmid. At 36 h after transfection, the cells were treated with oxygen glucose deprivation for 30 min. The cell lysates were subjected to immunoblot analysis with the indicated antibodies. Lower panel: the graph shows the results of the quantitative analysis of the P-JNK level (*n* = 3). **b** Upper panel: *Faf1*^+/+^ MEFs were transfected with VC or Flag-FAF1 plasmid (4 μg). At 36 h after transfection, the cells were treated with oxygen glucose deprivation for the indicated time periods. The cell lysates were immunoblotted with the indicated antibodies. Lower panel: the graph shows the results of the quantitative analysis of the P-JNK level (*n* = 3). **c** Upper panel: *Faf1*^+/+^ and *Faf1*^gt/gt^ MEFs were treated with oxygen glucose deprivation for the indicated times, and then, cell lysates were immunoblotted with the indicated antibodies. Lower panel: the graph shows the results of the quantitative analysis of the P-JNK level (*n* = 3). **d** Upper panel: *Faf1*^gt/gt^ MEFs were transfected with VC or Flag-FAF1 plasmid (4 μg). At 36 h after transfection, the cells were treated with oxygen glucose deprivation for the indicated times. The cell lysates were immunoblotted with the indicated antibodies. Lower panel: the graph shows the results of the quantitative analysis of the P-JNK level (*n* = 3). The data (**a** - **d**) are expressed as the mean ± S.E.M. of three independent experiments. Statistical comparisons were evaluated using ANOVA followed by Tukey’s HSD (**a**, **b** and **d**) and Dunnett’s T3 (**c**) post hoc analysis. ***P < 0.001, **P < 0.01, and *P < 0.05

Certain JNK1 interactors, such as peptidyl-prolyl isomerase 1 and JNK-interacting protein, enhance JNK1 activity [18, 50]. Based on the observed FAF1 binding to JNK1 upon ischemic stress, we investigated whether FAF1 activates JNK1 upon ischemic stress. We found that JNK1 phosphorylation was intensified by FAF1 overexpression in a dose- and time-dependent manner upon oxygen glucose deprivation insult in *Faf1*^+/+^ MEFs (Fig. 4a and b). In contrast, phosphorylated JNK1 was markedly reduced in *Faf1*^gt/gt^ MEFs compared with that in *Faf1*^+/+^ MEFs in response to oxygen glucose deprivation (Fig. 4c). Consistently, the phosphorylated form of c-Jun was also found to be significantly decreased in *Faf1*^gt/gt^ MEFs compared with that in *Faf1*^+/+^ MEFs upon ischemic stress (Additional file 5: Figure S4). Moreover, FAF1 reconstitution restored the JNK1 activation potential in *Faf1*^gt/gt^ MEFs (Fig. 4d). Next, we transfected truncated FAF1 mutants to *Faf1*^gt/gt^ MEFs. Upon oxygen glucose deprivation, the FAF1-DEDID transfection recovered the enzymatic activity of JNK1 in *Faf1*^gt/gt^ MEFs, whereas FAF1-FID and FAF1 C-term transfections did not (Additional file 6: Figure S5). These data indicate that FAF1-DEDID has the potential to activate JNK. Taken together, our data indicate that FAF1 plays an essential role in JNK1 activation upon oxygen glucose deprivation-induced ischemic stress.

### FAF1-mediated JNK1 activation triggers mitochondrial dysfunction upon ischemic stress

**Fig. 5:**
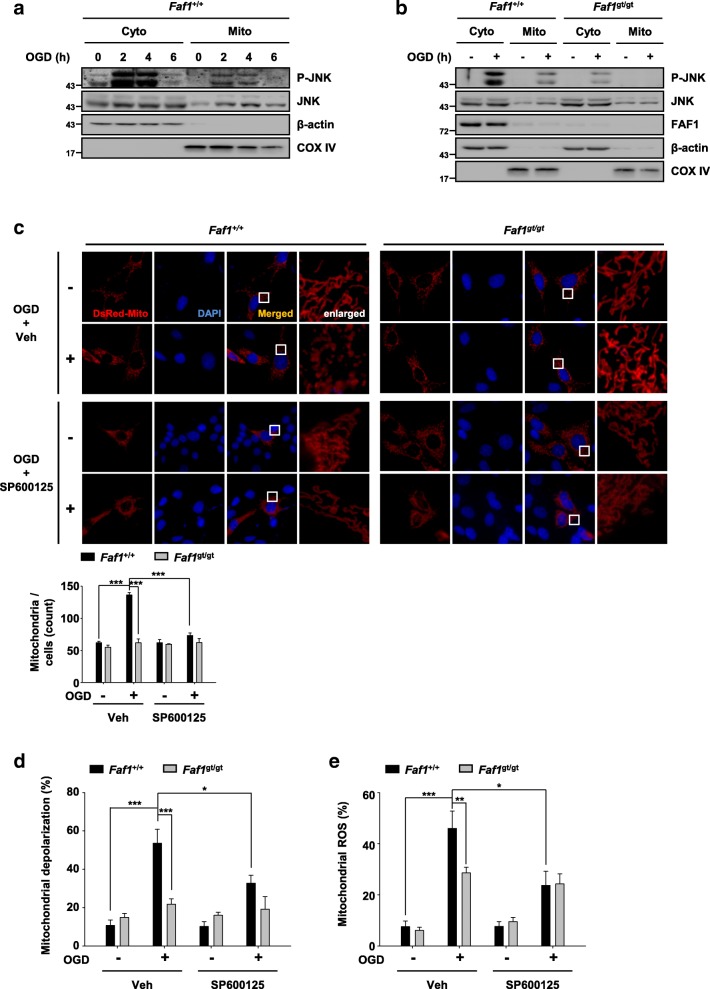
FAF1-mediated JNK1 activation triggers mitochondrial dysfunction upon ischemic stress. **a** *Faf1*^+/+^MEFs were treated with oxygen glucose deprivation for the indicated times and then fractionated into cytoplasmic and mitochondrial fractions. The fractions were analyzed by immunoblotting with anti-P-JNK, JNK1, anti-COX IV (mitochondrial marker) and anti-β-actin (cytoplasmic marker) antibodies. **b** *Faf1*^+/+^ and *Faf1*^gt/gt^ MEFs were treated with oxygen glucose deprivation for 2 h and then fractionated into cytoplasmic and mitochondrial fractions. The fractions were analyzed by immunoblotting with anti-P-JNK, JNK1, anti-COX IV and anti-β-actin antibodies. **c** Upper panel: *Faf1*^+/+^ and *Faf1*^gt/gt^ MEFs were transfected with DsRed-Mito plasmid for 48 h. Subsequently, the cells were treated with oxygen glucose deprivation in the presence or absence of 20 μM SP600125 for 2 h. Fluorescence images show the alterations in mitochondrial morphology*.* Scale bar = 20 μm. Bottom panel: the graph shows the quantification of mitochondrial fragmentation (*n* = 3). **d** *Faf1*^+/+^ and *Faf1*^gt/gt^ MEFs were untreated or treated with oxygen glucose deprivation in the presence or absence of 20 μM SP600125 for 5 h. Mitochondrial superoxide was stained using 10 μM MitoSOX reagent. The population of MitoSOX-positive cells was detected by flow cytometry. The graph shows the results of the quantitative analysis of MitoSOX-positive cells (*n* = 3). **e** *Faf1*^+/+^ and *Faf1*^gt/gt^ MEFs were untreated or treated with oxygen glucose deprivation in the presence or absence of 20 μM SP600125 for 5 h, and the mitochondrial potential was measured using a Muse analyzer. The graph was obtained from a quantitative analysis of depolarized cells (*n* = 3). The data (**c** - **e**) are expressed as the mean ± S.E.M. of three independent experiments. Statistical comparisons were evaluated using ANOVA followed by Tukey’s HSD (**c** - **e**) post hoc analysis. ***P < 0.001, **P < 0.01, and *P < 0.05

Translocation of JNK1 to mitochondria is a crucial event in necrotic cell death. Additionally, JNK1 phosphorylation is required for translocation to mitochondria [15, 16]. Therefore, we observed changes in the intracellular localization of phosphorylated JNK1 upon ischemic stress. In response to oxygen glucose deprivation, JNK1 was activated in a time-dependent manner for up to 4 h. In addition, the phosphorylation status of JNK1 returned to a resting level after 6 h (Additional file 7: Figure S6). Consistently, phosphorylated JNK1 translocated from the cytoplasm to the mitochondria for up to 4 h but not after 6 h in *Faf1*^*+*/+^ MEFs (Fig. 5a). However, translocation of phosphorylated JNK1 was attenuated in *Faf1*^gt/gt^ MEFs, indicating that FAF1 was required for JNK1 translocation (Fig. 5b). Therefore, we further questioned whether FAF1-mediated JNK1 translocation could cause mitochondrial damage in oxygen glucose deprivation-insulted MEFs. First, we examined the mitochondrial morphology in *Faf1*^*+*/+^ and *Faf1*^gt/gt^ MEFs. The mitochondrial morphology was shifted toward a fragmentation type upon oxygen glucose deprivation exposure in *Faf1*^*+*/+^ MEFs but not in *Faf1*^gt/gt^ MEFs (Fig. 5c). However, JNK1 inhibition induced by treatment with SP600125 in *Faf1*^*+*/+^ MEFs preserved the mitochondrial morphology under oxygen glucose deprivation stress, consistent with the finding that mitochondrial JNK1 is responsible for the disruption of mitochondrial morphology (Fig. 5c) [51]. Next, we measured the mitochondrial membrane potential and mitochondrial reactive oxygen species (ROS) level in *Faf1*^*+*/+^ and *Faf1*^gt/gt^ MEFs upon oxygen glucose deprivation. In response to oxygen glucose deprivation exposure, mitochondrial depolarization and mitochondrial ROS generation were significantly reduced in *Faf1*^gt/gt^ MEFs compared with those in *Faf1*^*+*/+^ MEFs (Fig. 5d and e). SP600125 also reduced mitochondrial ROS production and mitochondrial depolarization in *Faf1*^*+*/+^ MEFs (Fig. 5d and e). Collectively, our data show that FAF1-dependent JNK1 activation leads to mitochondrial dysfunction under ischemic conditions.

### FAF1 mediates JNK1-dependent necrosis in RGC5 cells upon ischemic stress

**Fig. 6:**
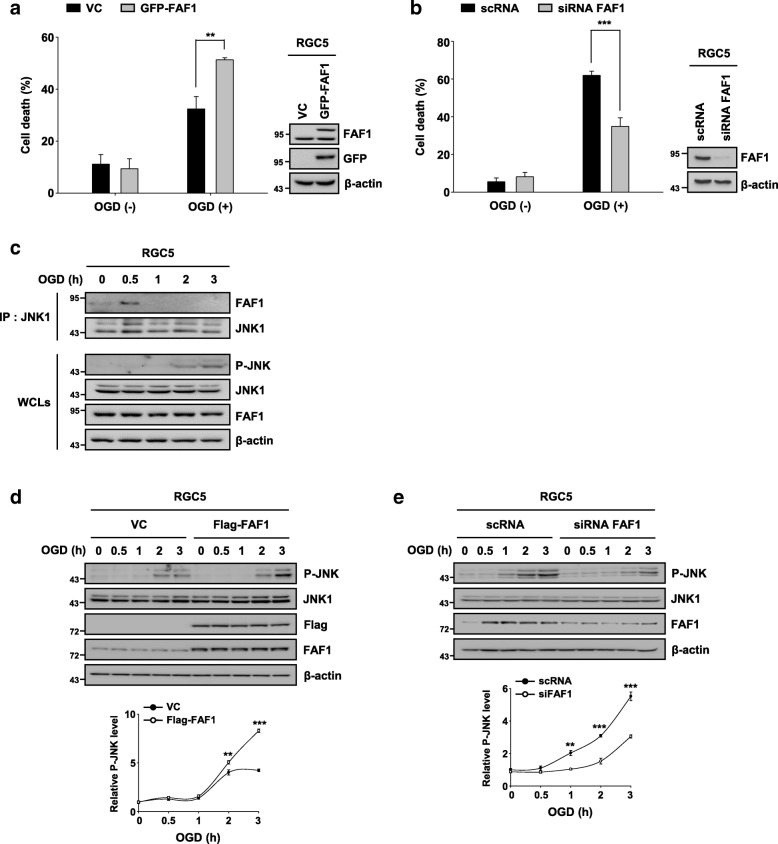
FAF1 mediates JNK1-dependent necrosis in RGC5 cells upon ischemic stress. **a** Left panel: RGC5 cells were transfected with VC or GFP-FAF1 plasmid (4 μg). At 48 h after transfection, the cells were treated with oxygen glucose deprivation for 8 h. Cell death was measured by flow cytometry (*n* = 3). Right panel: representative immunoblots showing the expression levels of FAF1, GFP and β-actin (**b**) Left panel: RGC5 cells were transfected with scrambled-siRNA (scRNA) or FAF1-targeting siRNA (siFAF1) for 48 h and then exposed to oxygen glucose deprivation for 10 h. Cell death was measured using flow cytometry (*n* = 3). Right panel: representative immunoblots showing the expression levels of FAF1 and β-actin. **c** RGC5 cells were treated with oxygen glucose deprivation for the indicated times. Whole cell lysates were immunoprecipitated with anti-JNK1 antibody, followed by an immunoblot assay with the indicated antibodies. **d** Upper panel: RGC5 cells were transfected with VC or Flag-FAF1 plasmid (2 μg). At 48 h after transfection, the cells were treated with oxygen glucose deprivation for the indicated times. Cell lysates were immunoblotted with the indicated antibodies. Lower panel: the graph shows the result of the quantitative analysis of the P-JNK level (*n* = 3). **e** Upper panel: RGC5 cells were transfected with scRNA or siFAF1. At 48 h post transfection, the cells were treated with oxygen glucose deprivation for the indicated times. Cell lysates were immunoblotted with the indicated antibodies. Lower panel: the graph shows the results of the quantitative analysis of the P-JNK level (*n* = 3). Data (**a**, **b**, **d**, and **e**) are expressed as the mean ± S.E.M. of three independent experiments. Statistical comparisons were evaluated using ANOVA followed by Tukey’s HSD (**a**, **b**, **d**, and **e**) post hoc analysis. ***P < 0.001, and **P < 0.01

Glaucoma results from ischemic injury caused by an elevation in IOP and is characterized by progressive loss of RGCs [52]. In addition, JNK1 is activated in human glaucoma patients as well as in various glaucoma animal models [12, 13]. Based on our data showing that FAF1 is a critical regulator of JNK1-dependent necrosis in MEFs upon ischemic stress, we investigated whether FAF1 is related to the death of RGC5 cells upon ischemic stress elicited by oxygen glucose deprivation. First, we examined whether FAF1 participates in the oxygen glucose deprivation-induced death of RGC5 cells. FAF1 overexpression enhanced oxygen glucose deprivation-induced death in a dose- and time-dependent manner in RGC5 cells (Fig. 6a, Additional file 8: Figure S7). In contrast, siRNA-mediated knockdown of FAF1 attenuated the death of RGC5 cells in response to oxygen glucose deprivation insult (Fig. 6b). These results indicate the involvement of FAF1 in oxygen glucose deprivation-induced death of RGC5 cells. Given that FAF1 interacts with JNK1 in MEFs upon ischemic stress, we examined the interaction between FAF1 and JNK1 in RGC5 cells. FAF1 also bound to JNK1 upon ischemic stress in RGC5 cells (Fig. 6c). Next, we examined whether FAF1 mediates JNK1 activation in RGC5 cells upon ischemic stress. JNK1 phosphorylation was intensified by FAF1 overexpression (Fig. 6d), whereas knockdown of FAF1 by siRNA reduced oxygen glucose deprivation-induced JNK1 activation in RGC5 cells (Fig. 6e). Together, these data demonstrate that FAF1 positively regulates JNK1 activity, consequently leading to RGC5 cell death upon ischemic stress.

### FAF1 ablation protects retinal cells in ganglion cell layer (GCL) against retinal ischemic injury in mouse model

**Fig. 7:**
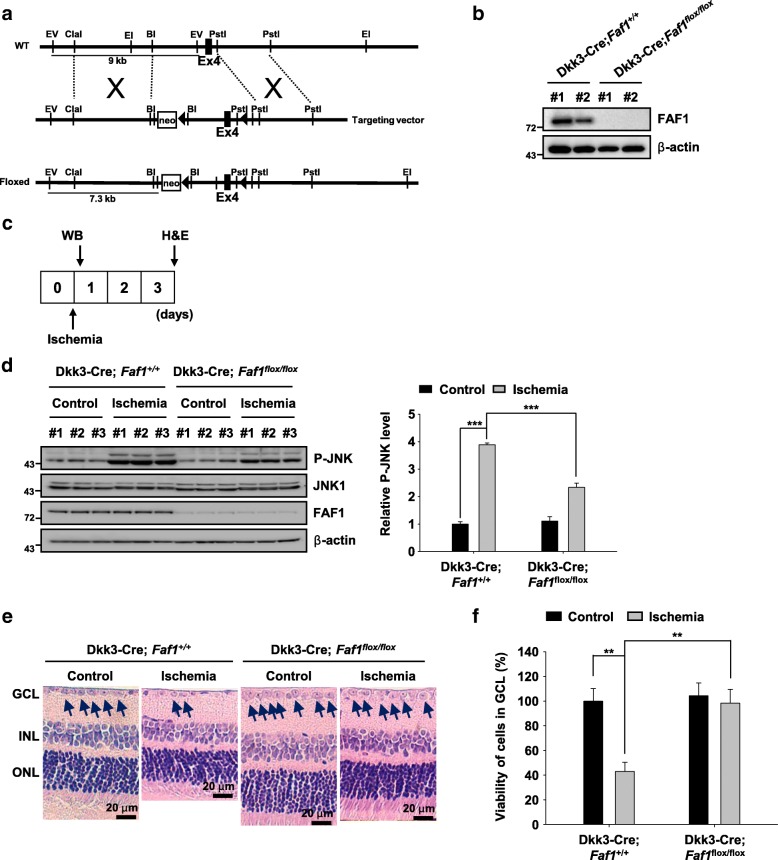
FAF1 ablation protects retinal cells in ganglion cell layer (GCL) against retinal ischemic injury in mouse model (**a**) Schematic representation of the strategy used to target the exons encoding FAF1. A fusion neo cassette was used for positive selection. The *EcoR*V (EV) restriction endonucleases used for DNA digestion: wild-type, 9 kb; neo loxP-floxed, 7 kb. Triangles: loxP sites. **b** FAF1 expression levels were assessed via immunoblot analysis of protein lysates from retinas of Dkk3-Cre;*Faf1*^*+/+*^ and Dkk3-Cre;*Faf1*^*flox/flox*^ mice. **c** Schematic diagram of the experimental design of the mouse model of retinal ischemia. The mice were sacrificed either 1 h or 3 days after induction of intraocular hypertension. **d** Left panel: immunoblot assay showing JNK1 activation in response to retinal ischemia in retinas of Dkk3-Cre;*Faf1*^*+/+*^ and Dkk3-Cre;*Faf1*^*flox/flox*^ mice (*n* = 3 eyes). Right panel: immunoblot assay showing JNK1 activation in response to retinal ischemia in retinas of Dkk3-Cre;*Faf1*^*+/+*^ and Dkk3-Cre;*Faf1*^*flox/flox*^ mice (*n* = 3 eyes). **e** The enucleated eyes were stained with HE. Histological images showing alterations in retinal morphology. INL, inner nuclear layer; ONL, outer nuclear layer. Scale bar = 20 μm. **f** The graph shows the number of cells in the GCL of the central region of the retina in HE-stained samples (*n* = 6 eyes). The data (**d** and **f**) are expressed as the mean ± S.E.M. of three independent experiments. Statistical comparisons were evaluated using ANOVA followed by Tukey’s HSD (**d** and **f**) post hoc analysis. ***P < 0.001, and **P < 0.01

Given that FAF1 functions as a key regulator of JNK1-dependent necrosis upon ischemic stress in RGC5 cells, we used a mouse model of retinal ischemia to validate the role of FAF1 in ischemia-induced neurodegeneration in vivo. FAF1-deleted embryos died at the two-cell stage [53], and analyses using the FAF1 hypomorphic mouse model (*Faf1*^gt/gt^) can be complicated due to the possible influence of neighboring tissues. Therefore, we created a FAF1 conditional knockout mouse with retina-specific deletion of *Faf1* using the Cre-loxP system. A strategic diagram of the development of the conditional knockout mouse is shown in Fig. 7a. The FAF1 expression levels were strongly diminished in the retinas of Dkk3-Cre;*Faf1*^flox/flox^ mice compared with those in the Dkk3-Cre;*Faf1*^+/+^ mice (Fig. 7b). Subsequently, we introduced ischemic damage to the retinas of Dkk3-Cre;*Faf1*^+/+^ and Dkk3-Cre;*Faf1*^flox/flox^ mice, as indicated in Fig. 7c. Retinal ischemia-induced JNK1 activation was observed in Dkk3-Cre;*Faf1*^+/+^ mice but not in Dkk3-Cre;*Faf1*^flox/flox^ mice (Additional file 9: Figure S8, Fig. 7d). This result was consistent with the data obtained in the cellular context (Figs. 4 and 6). Therefore, FAF1 is essential for JNK1 activation upon ischemic insult. Next, we performed a histological analysis using hematoxylin and eosin (HE) staining to investigate the effect of FAF1 on death of cells in GCL of ischemia-insulted eyes. Images of HE-stained retinal sections of ischemic eyes from Dkk3-Cre;*Faf1*^+/+^ mice showed decreased cells in GCL, suggesting degenerative changes in the ischemic eyes. In contrast, Dkk3-Cre;*Faf1*^flox/flox^ mice displayed resistance to ischemic cell death (Additional file 9: Figure S8, Fig. 7e). The ischemic retinas exhibited a 57.06 ± 6.91% reduction in cells in the GCL compared with non-ischemic contralateral retinas from Dkk3-Cre;*Faf1*^+/+^ mice. However, no significant difference was observed in the number of cells in GCL between ischemia-induced retinas and non-ischemic contralateral retinas of the Cre;*Faf1*^flox/flox^ mice (Fig. 7f). Collectively, our data show that FAF1 mediates retinal cell death in a mouse model of retinal ischemia.

## Discussion

Our previous study showed that FAF1 participates in oxidative stress-induced necrosis through PARP1 activation [31]. In this study, we elucidated a novel function of FAF1, which is a positive regulator of JNK1, in mediating ischemic stress-induced necrosis. Therefore, FAF1 is portrayed as a robust mediator of necrosis in dysregulated oxygen environments.

Because the depletion of cellular energy upon ischemic stress precludes caspase activation, necrosis predominates over apoptosis upon ischemic insult [54, 55]. Furthermore, regulated types of necrosis, including RIPK1-dependent necroptosis and PARP1-dependent parthanatos, play important roles in ischemia-reperfusion injury [45, 46]. Our data were also consistent in that necrosis was prevalent in MEFs and RGC5 cells in response to oxygen glucose deprivation. However, the apparent dispensability of catalytic activities of RIPK1 and PARP1 shows that regulated necroses are not primary contributors to oxygen glucose deprivation-induced necrosis. This finding contrasts with the apparent major role of regulated necroses in oxidative stress-induced cell death [56]. The discrepancy between the poor contribution of regulated necrosis in our study and the major role of regulated necrosis in other studies is intriguing. The cellular context of ischemic stress used in other studies often involves reperfusion, an oxidative stress, but our experimental condition excluded such oxidative stress. We speculate that this might explain the abovementioned difference. Overall, we report that a type of necrosis that is JNK1 dependent but hardly dependent on the RIPK1 and PARP1, chiefly contributes to ischemic cell death.

Many kinase inhibitors have been developed that compete for the ATP-binding site [57]. However, these ATP-competing inhibitors, including SP600125, are often non-specific kinase inhibitors. The protective potential of pharmacological inhibition using SP600125 was weaker than that of genetic ablation of JNK1. The weakening of the protective potential provided by SP600125 in Fig. 1f might be due to possible off-target effects of SP600125 on other survival kinases, such as AMP-activated protein kinase and phosphatidylinositol 3-kinase [58–60].

Interestingly, FAF1 takes differential paths depending on the oxygen level. FAF1 seems to choose an appropriate death pathway through alternative interactions with different partners depending on the cellular oxygen state. Similarly, p53, Bcl-2 and Bcl-X_L_ operate under different scenarios in response to cellular oxygen levels. p53 also switches partners depending on the oxygen level. In contrast, the cellular Bcl-2 and Bcl-X_L_ expression levels are altered according to the oxygen concentration. Therefore, the present findings add FAF1 to the list of proteins that function differentially to perform death-promoting roles depending on the surrounding oxygen concentration.

We are curious how FAF1 promotes JNK1 phosphorylation. Perhaps, interaction with FAF1 induces a conformational change in JNK1 to enhance autophosphorylation, similar to the phenomenon observed for prolyl isomerase, which converts JNK1 to its active form [50]. Another possibility is via an indirect method in which the interaction with FAF1 might facilitate interactions with other JNK1-activating factors. CK2 is known to regulate cell death through JNK1 activation in hepatocarcinoma cells and in embryonic hippocampal progenitor cells, and FAF1 can interact with CK2 [61]. Therefore, it is tempting to speculate that FAF1 recruits CK2 in close vicinity to JNK1 during oxygen glucose deprivation stress and subsequently permits JNK1 phosphorylation.

The antioxidant system is often impaired in glaucoma patients, leading to excessive ROS generation, which disrupts the trabecular meshwork [62]. Inefficient aqueous humor drainage via the impaired trabecular meshwork increases IOP. Subsequently, decreased blood flow triggers ischemic stress. Therefore, ischemic and oxidative stresses coexist in the pathological context of glaucoma [63]. The crucial role of FAF1 in both ischemic and oxidative stress-induced cell death [30, 31] highlights its essential contribution as a robust mediator of glaucoma progression.

Recent studies have indicated that autophagy is also responsible for RGC death and that JNK1 participates in ischemia/reperfusion-induced autophagic cell death through upregulation of Beclin-1 and downregulation of Bcl-2 [64, 65]. Given that FAF1 acts as a positive regulator of JNK1, FAF1 might regulate autophagic cell death as well as necrosis. Collectively, we suggest that developing a FAF1 inhibitor might offer a new opportunity for the treatment of various ischemic diseases.

## Conclusions

In conclusion, we previous reported that FAF1 functions critical role in oxidative stress-induced necrosis. In this study, we identified a novel role of FAF1 in mediating ischemic stress-induced necrosis. Therefore, FAF1 is a promising mediator of necrosis in dysregulated oxygen environments.

## Additional files

Additional file 1: **Table S1.** Primer sequences used for genotyping. (DOCX 14 kb)

Additional file 2: **Figure S1.** JNK1 contributes to necrosis in MEFs under other types of ischemic stress. (a and b) MEFs were treated with CH and TCZ for the indicated times. The type of cell death was determined by flow cytometry using double staining with annexin V and propidium iodide (PI). Annexin V-negative/PI-positive (upper left) cells represent necrotic cells, double-positive cells (upper right) represent late-stage apoptotic cells, and annexin V-positive/PI-negative cells (lower right) represent early-stage apoptotic cells. (c) MEFs were untreated or treated with CH for 10 h in the presence or absence of zVAD-fmk (50 μM), and cell death was determined by measuring PI uptake using flow cytometry (*n* = 3). (d) WT and Jnk1-/- MEFs were untreated or treated with CH for 10 h. Cell death was detected via flow cytometry (*n* = 3). (e) WT and Jnk1-/- MEFs were untreated or treated with TCZ for 12 h. Cell death was detected via flow cytometry (*n* = 3). (f) MEFs were pretreated with Nec-1 (50 uM) for 30 min and were then treated with TCZ for 12 h or CH for 10 h in the presence or absence of Nec-1 (50 μM). Cell death was determined by measuring PI uptake using flow cytometry (*n* = 3). The data (c-f) are expressed as the mean ± S.E.M. of three independent experiments. Statistical comparisons were performed using ANOVA followed by Dunnett’s T3 (c) and Tukey’s HSD (d - f) post hoc analysis. ***P < 0.001, and **P < 0.01. (PDF 79 kb)

Additional file 3: **Figure S2.** FAF1 is essential for necrosis upon application of other types of ischemic stress in MEFs. (a) Faf1+/+ and Faf1gt/gt MEF cells were treated with CH for the indicated times. Cell death was determined using flow cytometry (*n* = 3). (b) Faf1+/+ and Faf1gt/gt MEF cells were treated with TCZ for the indicated times. Cell death was determined using flow cytometry (*n* = 3). The data (a and b) are expressed as the mean ± S.E.M. of three independent experiments. Statistical comparisons were evaluated with ANOVA followed by Tukey’s HSD (a and b) post hoc analysis. ***P < 0.001, and *P < 0.05. (PDF 16 kb)

Additional file 4: **Figure S3.** Transfection of FAF1 and JNK1 did not cause cell death in HEK 293T cells under the experimental condition to evaluate the interaction between FAF1 and JNK1. HEK 293T cells were transfected with the indicated combinations of HA-FAF1 (1 ug) and Flag-JNK1 plasmids (1 ug). At 48 h after transfection, the cells were untreated or treated with oxygen glucose deprivation for 30 min. Cell death was determined by measuring PI uptake using a flow cytometer (*n* = 3). The data are expressed as the mean ± S.E.M. of three independent experiments. (PDF 5 kb)

Additional file 5: **Figure S4.** S4 FAF1 regulates JNK1-mediated activation of c-jun upon ischemic stress. (a) Upper panel: Faf1+/+ and Faf1gt/gt MEFs were treated with oxygen glucose deprivation for the indicated times. The cell lysates were immunoblotted with the indicated antibodies. (b) The graph shows the results of the quantitative analysis of the P-c-Jun level (*n* = 3). The data are expressed as the mean ± S.E.M. of three independent experiments. Statistical comparisons were evaluated using ANOVA followed by Dunnett’s T3 post hoc analysis. *P < 0.05. (PDF 73 kb)

Additional file 6: **Figure S5.** FAF1-DEDID activates JNK1 upon ischemic stress. (a) Upper panel: Faf1gt/gt MEFs were transfected with truncated FAF1 constructs. At 36 h after transfection, the cells were treated with oxygen glucose deprivation for 1 h, and cell lysates were then immunoblotted with the indicated antibodies. Lower panel: Schematic diagram of full length and truncated FAF1 constructs. (b) Faf1gt/gt MEFs were transfected with VC or Flag-FAF1-DEDID plasmid. At 36 h after transfection, the cells were treated with oxygen glucose deprivation for the indicated times. The cell lysates were immunoblotted with the indicated antibodies. (PDF 96 kb)

Additional file 7: **Figure S6.** Phosphorylated JNK1 reverts to resting levels after 6 h. Faf1+/+ were treated with oxygen glucose deprivation for the indicated times, and cell lysates were then immunoblotted with the indicated antibodies. (PDF 58 kb)

Additional file 8: **Figure S7.** FAF1 enhances necrosis in RGC5 cells upon ischemic stress induced by oxygen glucose deprivation. (a) Left panel: RGC5 cells were transfected with the indicated concentration of Flag-FAF1 plasmid. At 48 h after transfection, the cells were untreated or treated with oxygen glucose deprivation for 8 h. Cell death was determined by measuring PI uptake using a flow cytometer (*n* = 3). Right panel: representative immunoblots show the Flag, FAF1 and β-actin expression levels. (b) RGC5 cells were transfected with VC or Flag-FAF1 plasmids. At 48 h after transfection, the cells were treated with oxygen glucose deprivation for the indicated times. Cell death was determined by flow cytometry (*n* = 3). The data (a and b) are expressed as the mean ± S.E.M. of three independent experiments. Statistical comparisons were evaluated with ANOVA followed by Tukey’s HSD (a and b) post hoc analysis. ***P < 0.001, and **P < 0.01. (PDF 65 kb)

Additional file 9: **Figure S8.** JNK1 leads to cell death in GCL upon ischemic stress. (a) The mice were sacrificed 1, 6, and 24 h after induction of intraocular hypertension. Immunoblot assay showing JNK1 activation in response to retinal ischemia in retinas of Dkk3-Cre;Faf1+/+ mice (*n* = 3 eyes). (b) Immunoblot assay showing JNK1 activation in response to retinal ischemia in retinas of Dkk3-Cre;Faf1+/+ mice (*n* = 3 eyes). (c) The mice were sacrificed 1, 3, 5, and 7 day after induction of intraocular hypertension. The enucleated eyes were stained with HE. Histological images showing alterations in retinal morphology. (d) The graph shows the number of cells in the GCL of the central region of the retina in HE-stained samples (*n* = 5 eyes). The data (b and d) are expressed as the mean ± S.E.M. of three independent experiments. Statistical comparisons were evaluated with ANOVA followed by Tukey’s HSD (b and d) post hoc analysis. ***P < 0.001, **P < 0.01, and *P < 0.05. (PDF 117 kb)

## Abbreviations

FAF1: Fas-associated factor 1
GCL: Ganglion cell layer
HE: Hematoxylin and eosin
IOP: Intraocular pressure
JNK: c-Jun-N-terminal kinase
Mcl-1: Myeloid cell leukemia sequence 1
MEF: Mouse embryonic fibroblast
PARP1: Poly(ADP-ribose)polymerase 1
PI: Propidium iodide
RGC: Retinal ganglion cell
ROS: Reactive oxygen species
Sab: SH3BP5
STS: Staurosporine
