# Supplementary material for: FAF1 mediates necrosis through JNK1-mediated mitochondrial dysfunction leading to retinal degeneration in the ganglion cell layer upon ischemic insult
Source: Cell Commun Signal. 2018 Sep 10;16:56. doi: 10.1186/s12964-018-0265-7 (PMC6131785; doi:10.1186/s12964-018-0265-7)
Supplement: Supplementary file 1 — Table S1. Primer sequences used for genotyping. (DOCX 14 kb) [file 12964_2018_265_MOESM1_ESM.docx]

**Table S1. Primer sequences used for** **genotyping**

Tail DNA was used for genomic PCR to determine the genotype of each mouse. The primer sequences used to determine the genotype were as follows:

| **Primer** | **Sequence (5´ → 3´)** |
| --- | --- |
| FAF1 Forward | TTG TGC TGC CAC AAA AGA CT |
| FAF1 Reverse | AAG CAA GTC CAT CCC TTT TG |
| Cre Forward | GCG GTC TGG CAG TAA AAA CT |
| Cre Reverse | GTG AAA CAG CAT TGC TGT CA |
