# Supplementary figures and images for: FAF1 mediates necrosis through JNK1-mediated mitochondrial dysfunction leading to retinal degeneration in the ganglion cell layer upon ischemic insult
Source: Cell Commun Signal. 2018 Sep 10;16:56. doi: 10.1186/s12964-018-0265-7 (PMC6131785; doi:10.1186/s12964-018-0265-7)

# Supplementary Figure S1.

a

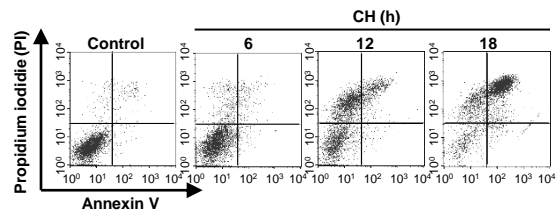

b

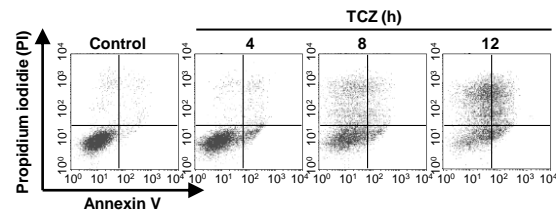

c

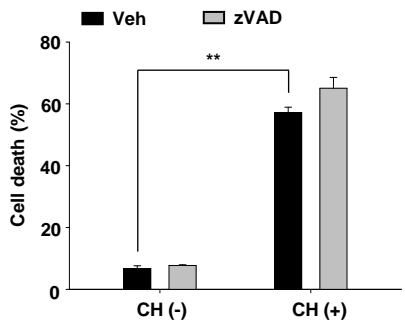

d

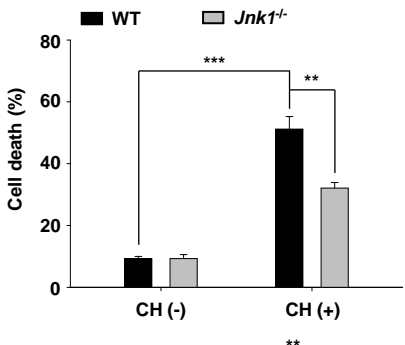

e

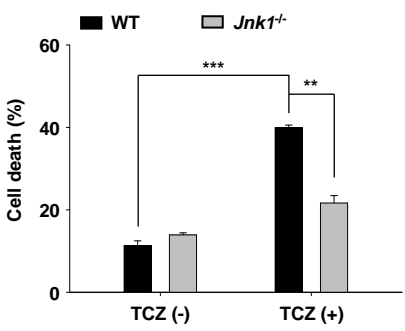

f

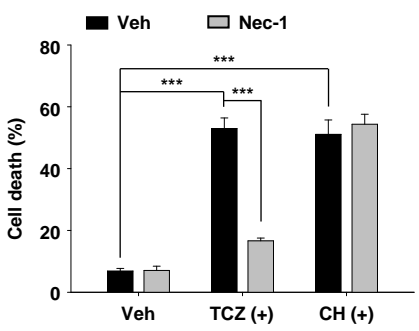

Supplement: Supplementary file 2 — Figure S1. JNK1 contributes to necrosis in MEFs under other types of ischemic stress. (a and b) MEFs were treated with CH and TCZ for the indicated times. The type of cell death was determined by flow cytometry using double staining with annexin V and propidium iodide (PI). Annexin V-negative/PI-positive (upper left) cells represent necrotic cells, double-positive cells (upper right) represent late-stage apoptotic cells, and annexin V-positive/PI-negative cells (lower right) represent early-stage apoptotic cells. (c) MEFs were untreated or treated with CH for 10 h in the presence or absence of zVAD-fmk (50 μM), and cell death was determined by measuring PI uptake using flow cytometry (n = 3). (d) WT and Jnk1-/- MEFs were untreated or treated with CH for 10 h. Cell death was detected via flow cytometry (n = 3). (e) WT and Jnk1-/- MEFs were untreated or treated with TCZ for 12 h. Cell death was detected via flow cytometry (n = 3). (f) MEFs were pretreated with Nec-1 (50 uM) for 30 min and were then treated with TCZ for 12 h or CH for 10 h in the presence or absence of Nec-1 (50 μM). Cell death was determined by measuring PI uptake using flow cytometry (n = 3). The data (c-f) are expressed as the mean ± S.E.M. of three independent experiments. Statistical comparisons were performed using ANOVA followed by Dunnett’s T3 (c) and Tukey’s HSD (d - f) post hoc analysis. ***P < 0.001, and **P < 0.01. (PDF 79 kb) [file 12964_2018_265_MOESM2_ESM.pdf]

# Supplementary Figure S2.

a

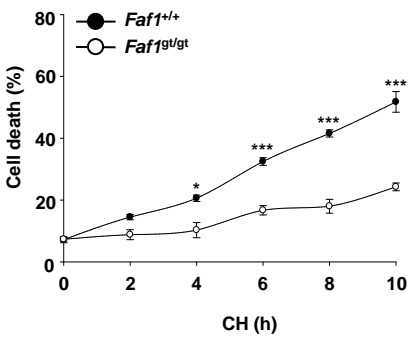

b

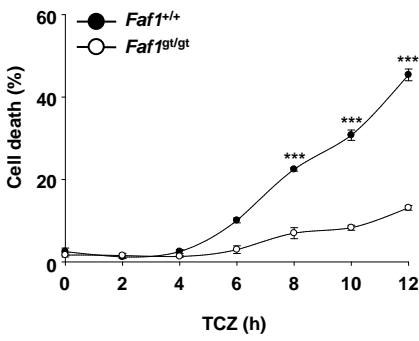

Supplement: Supplementary file 3 — Figure S2. FAF1 is essential for necrosis upon application of other types of ischemic stress in MEFs. (a) Faf1+/+ and Faf1gt/gt MEF cells were treated with CH for the indicated times. Cell death was determined using flow cytometry (n = 3). (b) Faf1+/+ and Faf1gt/gt MEF cells were treated with TCZ for the indicated times. Cell death was determined using flow cytometry (n = 3). The data (a and b) are expressed as the mean ± S.E.M. of three independent experiments. Statistical comparisons were evaluated with ANOVA followed by Tukey’s HSD (a and b) post hoc analysis. ***P < 0.001, and *P < 0.05. (PDF 16 kb) [file 12964_2018_265_MOESM3_ESM.pdf]

# Supplementary Figure S3.

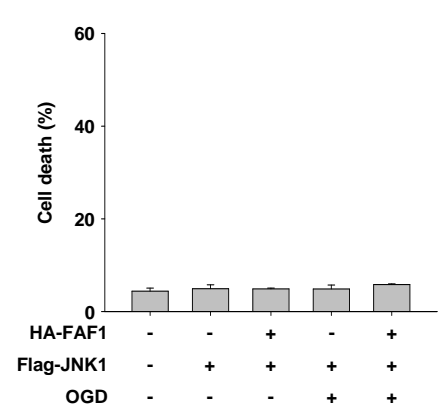

Supplement: Supplementary file 4 — Figure S3. Transfection of FAF1 and JNK1 did not cause cell death in HEK 293T cells under the experimental condition to evaluate the interaction between FAF1 and JNK1. HEK 293T cells were transfected with the indicated combinations of HA-FAF1 (1 ug) and Flag-JNK1 plasmids (1 ug). At 48 h after transfection, the cells were untreated or treated with oxygen glucose deprivation for 30 min. Cell death was determined by measuring PI uptake using a flow cytometer (n = 3). The data are expressed as the mean ± S.E.M. of three independent experiments. (PDF 5 kb) [file 12964_2018_265_MOESM4_ESM.pdf]

# Supplementary Figure S4.

a

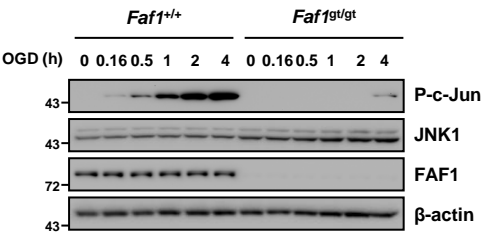

b

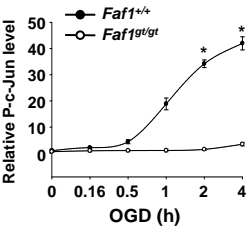

Supplement: Supplementary file 5 — Figure S4. S4 FAF1 regulates JNK1-mediated activation of c-jun upon ischemic stress. (a) Upper panel: Faf1+/+ and Faf1gt/gt MEFs were treated with oxygen glucose deprivation for the indicated times. The cell lysates were immunoblotted with the indicated antibodies. (b) The graph shows the results of the quantitative analysis of the P-c-Jun level (n = 3). The data are expressed as the mean ± S.E.M. of three independent experiments. Statistical comparisons were evaluated using ANOVA followed by Dunnett’s T3 post hoc analysis. *P < 0.05. (PDF 73 kb) [file 12964_2018_265_MOESM5_ESM.pdf]

Supplementary Figure S5.

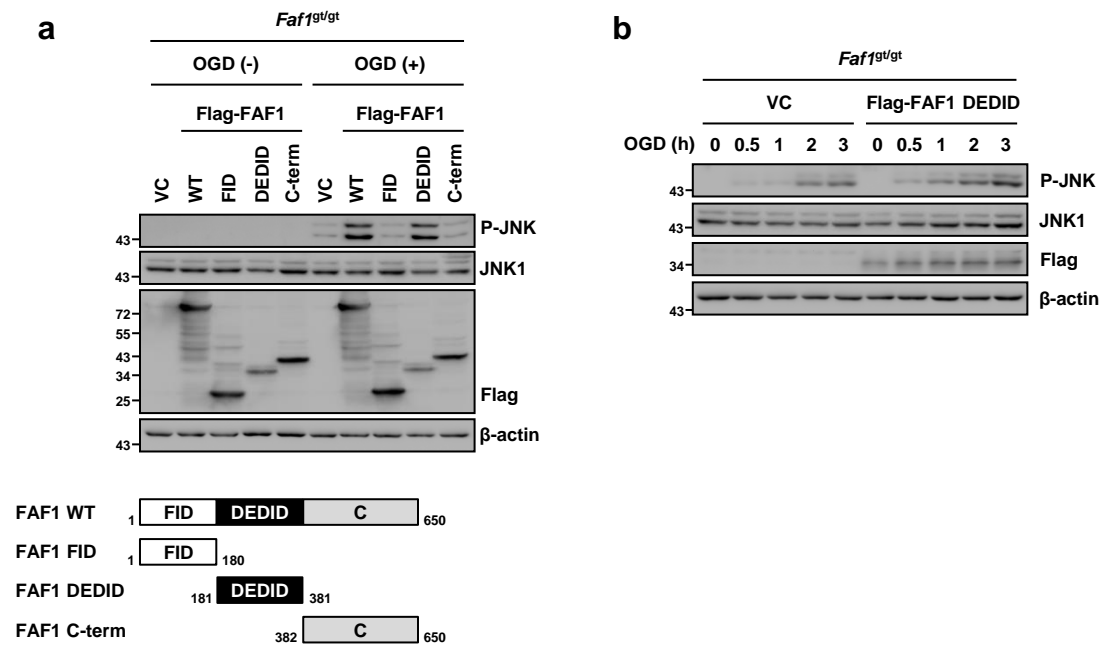

Supplement: Supplementary file 6 — Figure S5. FAF1-DEDID activates JNK1 upon ischemic stress. (a) Upper panel: Faf1gt/gt MEFs were transfected with truncated FAF1 constructs. At 36 h after transfection, the cells were treated with oxygen glucose deprivation for 1 h, and cell lysates were then immunoblotted with the indicated antibodies. Lower panel: Schematic diagram of full length and truncated FAF1 constructs. (b) Faf1gt/gt MEFs were transfected with VC or Flag-FAF1-DEDID plasmid. At 36 h after transfection, the cells were treated with oxygen glucose deprivation for the indicated times. The cell lysates were immunoblotted with the indicated antibodies. (PDF 96 kb) [file 12964_2018_265_MOESM6_ESM.pdf]

# Supplementary Figure S6.

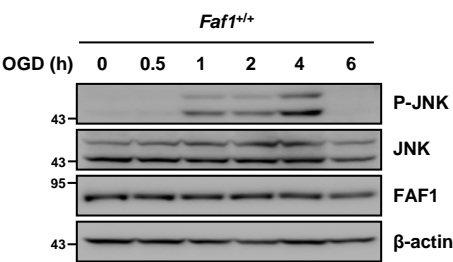

Supplement: Supplementary file 7 — Figure S6. Phosphorylated JNK1 reverts to resting levels after 6 h. Faf1+/+ were treated with oxygen glucose deprivation for the indicated times, and cell lysates were then immunoblotted with the indicated antibodies. (PDF 58 kb) [file 12964_2018_265_MOESM7_ESM.pdf]

# Supplementary Figure S7.

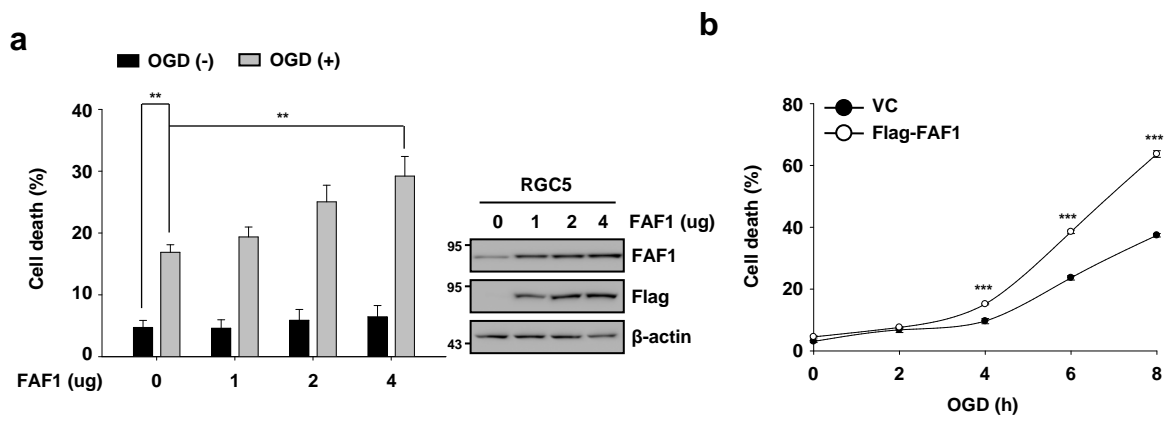

Supplement: Supplementary file 8 — Figure S7. FAF1 enhances necrosis in RGC5 cells upon ischemic stress induced by oxygen glucose deprivation. (a) Left panel: RGC5 cells were transfected with the indicated concentration of Flag-FAF1 plasmid. At 48 h after transfection, the cells were untreated or treated with oxygen glucose deprivation for 8 h. Cell death was determined by measuring PI uptake using a flow cytometer (n = 3). Right panel: representative immunoblots show the Flag, FAF1 and β-actin expression levels. (b) RGC5 cells were transfected with VC or Flag-FAF1 plasmids. At 48 h after transfection, the cells were treated with oxygen glucose deprivation for the indicated times. Cell death was determined by flow cytometry (n = 3). The data (a and b) are expressed as the mean ± S.E.M. of three independent experiments. Statistical comparisons were evaluated with ANOVA followed by Tukey’s HSD (a and b) post hoc analysis. ***P < 0.001, and **P < 0.01. (PDF 65 kb) [file 12964_2018_265_MOESM8_ESM.pdf]

# Supplementary Figure S8.

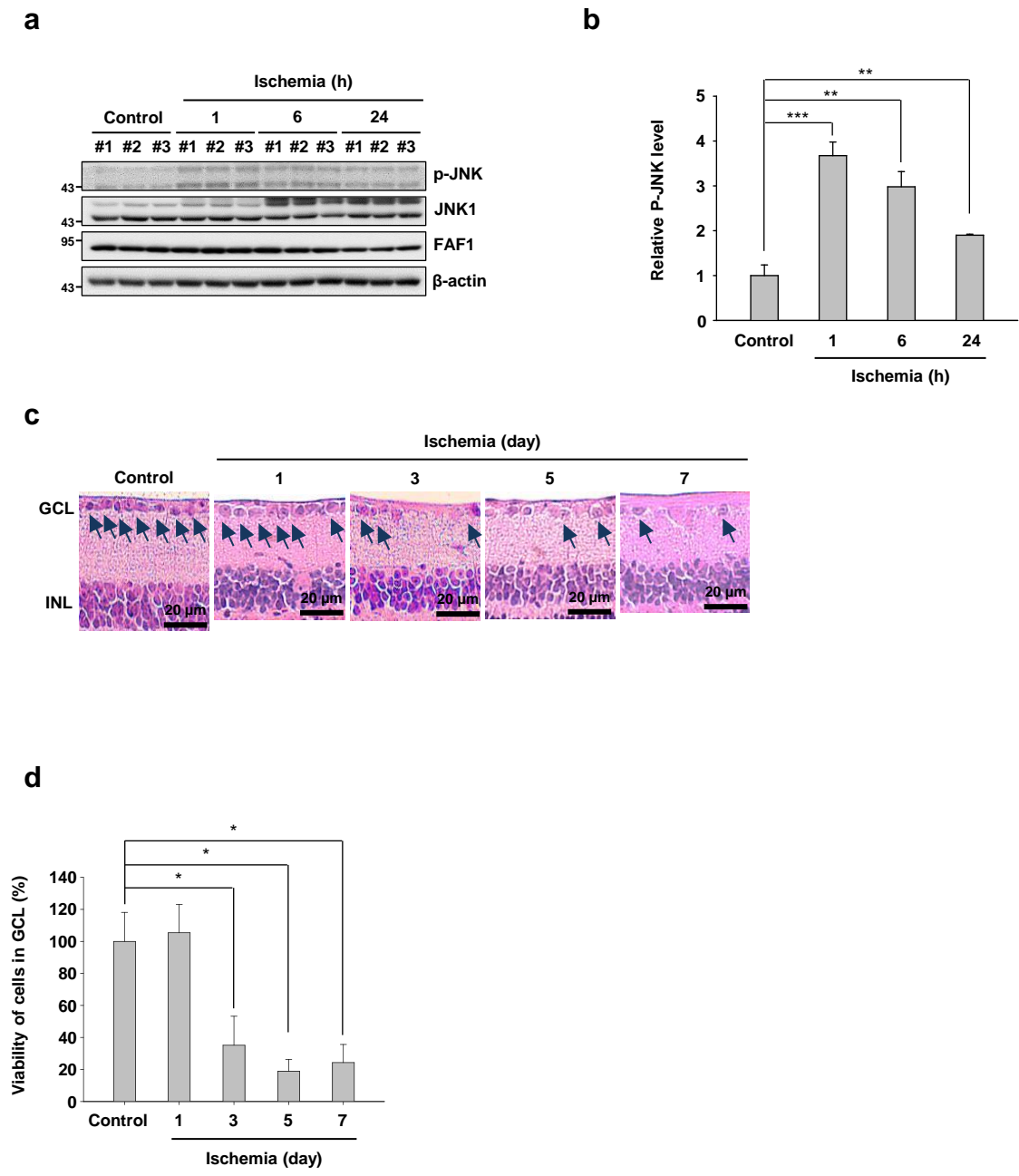

Supplement: Supplementary file 9 — Figure S8. JNK1 leads to cell death in GCL upon ischemic stress. (a) The mice were sacrificed 1, 6, and 24 h after induction of intraocular hypertension. Immunoblot assay showing JNK1 activation in response to retinal ischemia in retinas of Dkk3-Cre;Faf1+/+ mice (n = 3 eyes). (b) Immunoblot assay showing JNK1 activation in response to retinal ischemia in retinas of Dkk3-Cre;Faf1+/+ mice (n = 3 eyes). (c) The mice were sacrificed 1, 3, 5, and 7 day after induction of intraocular hypertension. The enucleated eyes were stained with HE. Histological images showing alterations in retinal morphology. (d) The graph shows the number of cells in the GCL of the central region of the retina in HE-stained samples (n = 5 eyes). The data (b and d) are expressed as the mean ± S.E.M. of three independent experiments. Statistical comparisons were evaluated with ANOVA followed by Tukey’s HSD (b and d) post hoc analysis. ***P < 0.001, **P < 0.01, and *P < 0.05. (PDF 117 kb) [file 12964_2018_265_MOESM9_ESM.pdf]
